# Evaluation of Sarcopenia in Renal Transplant Recipients

**DOI:** 10.5812/numonthly.20055

**Published:** 2014-07-05

**Authors:** Nihal Ozkayar, Bulent Altun, Meltem Halil, Mehmet E. Kuyumcu, Gunes Arik, Yusuf Yesil, Tolga Yildirim, Rahmi Yilmaz, Servet Ariogul, Cetin Turgan

**Affiliations:** 1 Department of Nephrology, Ankara Numune Education and Research Hospital, Ankara, Turkey; 2 Department of Nephrology, Faculty of Medicine, Hacettepe University, Ankara, Turkey; 3 Division of Geriatric Medicine, Faculty of Medicine, Hacettepe University, Ankara, Turkey

**Keywords:** Muscle Strength, Renal Transplantation, Sarcopenia

## Abstract

**Background:**

Chronic kidney disease can lead to sarcopenia; however, no study has described sarcopenia in the patients undergoing renal transplantation.

**Objectives:**

The aim of the present study was to assess the prevalence of sarcopenia in renal transplant recipients (RTR) and to evaluate the demographic and metabolic risk factors associated with sarcopenia in these patients.

**Patients and Methods:**

Sarcopenia was diagnosed by measuring handgrip strength in 166 RTR (68 females and 98 males; mean age, 37.9 ± 11.9 years). Basal metabolic rate, fat mass, free-fat mass, total body water, body mass index, and calf circumference were determined, along with blood biochemistry, vitamin D levels, and glomerular filtration rate.

**Results:**

Among 166 patients, sarcopenia was present in 34 (20.5%). Handgrip, basal metabolic rate, free fat mass, and total body water were significantly lower in patients with sarcopenia in comparison with those without sarcopenia. There were no differences between patients with and without sarcopenia in terms of mean time since transplantation, the presence of diabetes mellitus, hypertension, coronary artery disease, hyperlipidemia, glomerular filtration rate, and body mass index. Univariate analysis revealed significant differences between patients with and without sarcopenia with respect to age (mean of 43.70 ± 13.97 and 36.37 ± 10.82 years, respectively; P = 0.007) and 25-OH vitamin D levels (median (IQR) of 12 (2-39) and 17.70 (3-68) μg/L, respectively; P = 0.024). There was a statistically significant positive correlation between vitamin D levels and handgrip strength (r = 0.334; P < 0.001). Multivariate regression analysis determined that age was an independent predictive variable of sarcopenia in RTR (β = 1.060; 95% CI, 1.017-1.105; and P = 0.006).

**Conclusions:**

Chronic renal disease contributes to sarcopenia, which may develop at an earlier age in RTR.

## 1. Background

Sarcopenia is the progressive generalized loss of skeletal muscle mass, strength, and function. Although sarcopenia is essentially a disorder of advanced age, it can also occur at younger ages secondary to chronic nutritional problems, chronic diseases, malignancies, low level of physical activity, some drugs side effects (1, 2). Chronic kidney disease (CKD) can lead to sarcopenia. The National Health and Nutrition Examination Survey (NHANES) III study on the association between kidney function and sarcopenia reported that the prevalence of sarcopenia increases as the glomerular filtration rate (GFR) decreases (3). Furthermore, the loss of muscular mass may occur earlier and more markedly in patients with CKD than in others of the same age (4).

Diagnosis of sarcopenia is important, as it can lead to physical disability, low quality of life, falls, increased risk of fracture, and even death (5, 6). Muscular strength, which is used to evaluate sarcopenia via handgrip strength (HGS), is a repeatable, low-cost, and simple measurement of muscular strength in clinical practice (7). Although the lower extremities are more closely associated with physical function, research has shown that HGS correlates with extremity muscle force, knee stretch moment, and calf sectional muscle. In addition, low HGS is more closely correlated with impaired mobility and clinical outcome, than is, low muscular mass (6).

Although some studies have investigated sarcopenia in CKD, to the best of our knowledge none have examined the presence of sarcopenia in renal transplant recipients (RTR).

## 2. Objectives

The aim of our study was to investigate the presence of sarcopenia in RTR based on HGS measurement and to evaluate the demographic and metabolic risk factors of sarcopenia in these patients.

## 3. Patients and Methods

### 3.1. Patient Characteristics

This cross-sectional study included 166 RTR that were referred to the outpatient clinic of Division of Nephrology, Department of Internal Medicine, Hacettepe University Hospital, Ankara, Turkey. The study was conducted between March 2012 and May 2012. Inclusion criteria were renal transplantation, age > 18 years, equal to or more than three months since transplantation, and a GFR > 25 mL/min/1.73 m^2^. Patients who were unable to communicate with the researchers, were diagnosed with malignancy prior to the study, or had arthritis or neuromuscular diseases involving the hands bilaterally, congestive heart failure/nephrotic syndrome with severe edema, a pacemaker or prosthesis, or severe electrolyte imbalance were excluded, as these factors could interfere with accurate HGS measurement and bioimpedance analysis. The study protocol was approved by the Hacettepe University Ethics Committee and was performed in accordance with the Declaration of Helsinki. All the participants provided written informed consent to participate.

### 3.2. Study Procedures

Height was measured to the nearest 0.5 cm via a stadiometer with patients barefoot. Body weight was measured using a bioimpedance analysis (BIA) device (TBF-300 body composition analyzer, Tanita, IL, USA) with patients in light clothing. Basal metabolic rate (BMR), fat mass (FM), free fat mass (FFM), resistance, and total body water (TBW) were measured via BIA. The body mass index (BMI) was calculated as body weight divided by squared height (kg/m^2^). Calf circumference (CC), considered by the World Health Organization (WHO) to be the most sensitive anthropometric measure of muscle mass in the elderly, was measured using a standard anthropometric tape with the participants in a standing position. The tape was wrapped around the widest part of the calf of the nondominant leg to obtain the maximal circumference; subcutaneous tissues were not compressed (8).

HGS was evaluated using a Takei TKK 5401 digital handgrip dynamometer (Takei Scientific Instruments Co., Ltd, Niigata, Japan); maximum strength of the dominant hand was measured three times and the highest recorded value was considered maximal grip strength. Several methods for diagnosing sarcopenia based on HGS have been reported. In the present study, poor grip strength was defined according to Cardiovascular Health Study CHS (CardiovascularHealth Study) criteria (7). In males with a BMI ≤ 24 kg/m^2^, 24.1 to 28 kg/m^2^, and > 28 kg/m^2^ the cutoff point for HGS was 29, 30, and 32 kg, respectively. In females with a BMI ≤ 23 kg/m2, 23.1 to 26 kg/m^2^, 26.1 to 29 kg/m^2^, and > 29 kg/m^2^ the cutoff for HGS was 17, 17.3, 18, and 21 kg, respectively.

Blood samples were obtained from the antecubital vein between 08:30 and 10:00 AM, following fasting of≥ 8 hours. A hospital auto-analyzer was used to measure the complete blood count, kidney function tests, calcium, potassium, total protein, albumin, fasting plasma glucose, lipid profile, and 24-hour protein excretion. Measurement of 25-OH vitamin D was performed via high-performance liquid chromatography and parathyroid hormone (PTH) was measured via immunoradiometric assay. GFR was calculated using the CKD-EPI (Chronic Kidney Disease Epidemiology Collaboration) equation, as follows: GFR = 141 × min (_Serum _creatinine /κ,1)^α^ × max (_Serum _creatinine /κ,1)^-1.209 ^× 0.993 Age × 1.018 [in females] × 1.159 [in blacks] (9).

### 3.3. Statistical Analysis

All statistical analyses were performed using SPSS v.17.0 for Windows (SPSS Inc, Chicago, IL, USA). The Kolmogorov-Smirnov test was used to determine distribution of characteristics and the Levene’s test was used to determine the homogeneity of variances. Continuous variables were expressed as mean ± standard deviation (SD), or median and interquartile range (IQR), according to distribution characteristics. Categorical variables were expressed as number and percentage. Continuous variables were compared using the t test or Mann-Whitney U test, as appropriate. Categorical variables were compared using the Chi squared test. Pearson’s correlation coefficient was used for continuous variables with normal distribution and Spearman’s correlation coefficient was used for continuous variables not normally distributed. Logistic regression analysis was used to determine the effect of sarcopenia-related parameters on development of sarcopenia. The level of statistical significance was set at P < 0.05.

## 4. Results

In total, 166 RTR who met the inclusion criteria (68 females, 98 males) were enrolled in the study. Mean age of the patients was 37.9 ± 11.9 years. Overall, 34 patients (20.5%) had sarcopenia. The patients’ demographic data and laboratory findings are shown in Table 1.

Handgrip, BMR, FFM, and TBW were significantly lower in the patients with sarcopenia than in those without sarcopenia (Table 1). There were no differences in mean time since transplantation, the presence of diabetes mellitus, hypertension, and coronary artery disease, GFR, BMI, total cholesterol, and low-density lipoprotein levels between patients with and without sarcopenia (Table 1). Immunosuppressive treatment protocols were similar in patients.

According to univariate analysis, there were significant differences between the patients with and without sarcopenia with respect to age (mean of 43.70 ± 13.97 and 36.37 ± 10.82 years, respectively; P = 0.007), 25-OH vitamin D levels (median [IQR] of 12 [2-39] and 17.70 [3-68] μg/L, respectively; P = 0.024), and triglyceride (mean of 178.58 ± 58.24 and 190.02 ± 34.29 mg/dL, respectively; P = 0.039). 

**Figure 1.**
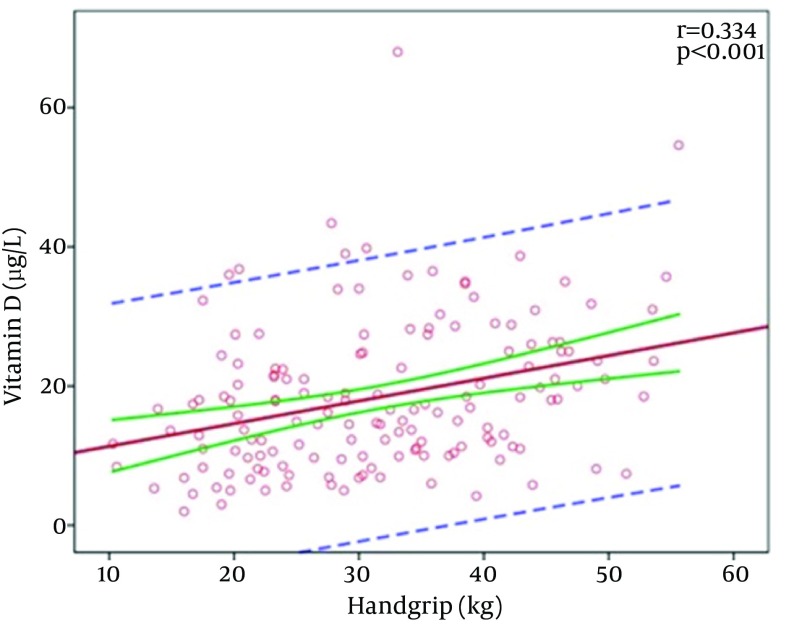
The vitamin D Level and Handgrip Strength

There was a significant positive correlation between the vitamin D level and handgrip strength (r = 0.334; P < 0.001) (Figure 1).

All of the above parameters were used in multivariate analysis. The results of multiple logistic regression analysis of the possible correlates for sarcopenia are summarized in Table 2. Multivariate regression analysis showed that age was an independent variable that predicted the occurrence of sarcopenia in RTR (β = 1.060; 95% CI, 1.017-1.105; and P = 0.006).

**Table 1.** Demographic and Laboratory Characteristics of Patients Regarding Sarcopenia ^a,b^

Parameters	Patients With Sarcopenia (n = 34)	Patients Without Sarcopenia (n = 132)	P Value
**Age, y**	43.70 ± 13.97	36.37 ± 10.82	0.007
**Gender, No. (Male/Female)**	22/12	75/56	0.431
**Posttransplantation Time, mo**	35.70 ± 6.27	33.00 ± 3.71	0.148
**BMI, kg/m** ^**2**^	25.50 ± 6.35	24.68 ± 4.76	0.502
**Handgrip, kg**	21.51 ± 6.35	33.69 ± 9.87	< 0.001
**CC, cm**	34.80 ± 4.25	36.00 ± 3.89	0.123
**Skeletal Muscle Mass Index, kg/m** ^**2**^	10.94 ± 4.21	10.18 ± 2.22	0.335
**BMR, kcal**	1437.71 ± 186.37	1567.19 ± 238.32	0.002
**FM, kg**	15.76 ± 10.70	15.22 ± 9.84	0.784
**FFM, kg**	49.24 ± 8.40	54.02 ± 10.54	0.018
**TBW, kg**	36.05 ± 6.14	39.55 ± 7.71	0.018
**GFR, mL/min/1.73 m** ^**2**^	61.02 (23-115)	61 (19-119)	0.589
**Vitamin D, μg/L**	12 (2-39)	17.70 (3-68)	0.024
**Albumin, g/dL**	4.10 ± 0.42	4.59 ± 1.40	0.401
**PTH, pg/mL**	57 (23-271)	74.60 (3-444)	0.938
**TC, mg/dL**	178.58 ± 58.24	190.02 ± 34.29	0.279
**LDL, mg/dL**	104.58 ± 40.71	111.82 ± 27.15	0.332
**TG, mg/dL**	129.02 ± 57.82	160.85 ± 84.09	0.039
**Proteinuria, mg/d**	183.50 (50.00-4990.00)	160.50 (11.00-5300.00)	0.410
**HT, No. (%)**	25 (73.5)	85 (64.4)	0.315
**DM**	7 (21.2)	17 (12.9)	0.225
**CAD**	2 (5.9)	5 (3.8)	0.594

^a^ Data are presented as mean ± SD, median (interquartile range), or No. (%).

^b^ Abbreviations: BMI, body mass index; CC, calf circumference; BMR, basal metabolism rate; FM, fat mass; FFM, free fat mass; TBW, total body water; GFR, glomerular filtration rate; PTH, parathormone; TC, total cholesterol; LDL, low-density lipoprotein; TG, triglyceride; HT, hypertension; DM, diabetes mellitus; and CAD, coronary artery disease.

**Table 2.** The Multiple Logistic Regression Analysis of the Possible Correlates for Sarcopenia ^a^

Parameters	Univariate				Multivariate			
	β (OR)	95% CI		P	β (OR)	95% CI		P
		Lower	Upper			Lower	Upper	
**Age, y**	1.054	1.020	1.089	0.002	1.060	1.017	1.105	0.006
**FFM, kg**	0.951	0.911	0.992	0.021	-	-	-	-
**TBW, kg**	0.933	0.880	0.989	0.021	-	-	-	-
**Vitamin D, μg/L**	0.953	0.910	0.998	0.043	-	-	-	-
**TG, mg/dL**	0.993	0.987	0.998	0.038	-	-	-	-
**BMR, kcal**	0.997	0.995	0.999	0.006	-	-	-	-

^a^ Abbreviations: β, exponentiated logistic coefficients; OR, odds ratio; FFM, free fat mass; TBW, total body water; TG, triglyceride; and BMR, basal metabolic rate.

## 5. Discussion

The present study determined the prevalence of sarcopenia in RTR. Our findings indicated that sarcopenia occurred in the RTR at a younger age in comparison with the general population. In the elderly, sarcopenia is associated with functional impairment, cardiopulmonary failure, and physical disability. Patients with sarcopenia also have an increased risk of hospitalization and a high mortality rate (10, 11). Although it is known that sarcopenia is essentially a disease of advanced age and may coincide with various diseases in younger patients, there is a lack of information on the development of sarcopenia in RTR.

The prevalence of sarcopenia varies widely in elderly cohorts due to the lack of a consensus definition of sarcopenia and differences in diagnostic criteria including assessment of muscle mass, muscle strength, and physical performance (12). The prevalence of sarcopenia is 5% to 13% in patients aged 60 to 70 years, whereas it ranges from 11% to 50% in patients older than 80 years (13). Although there are few studies on the prevalence of sarcopenia in patients younger than 60 years of age, it was reported that the prevalence was 0% to 20.8% in males and 0% to 25.8% in females of this age group (12).

Many factors lead to the development and progression of sarcopenia including advanced age, bedridden state, sedentary lifestyle, insulin resistance, advanced organ failure (heart, lung, liver, or kidney), inflammatory diseases, malignancies, malabsorption, and drugs side effects (14-16). The most important risk factors for the developing sarcopenia are advanced age and female sex (17-19). In the present study, patients experienced sarcopenia at younger age than those reported earlier. In addition, no association was observed between sarcopenia and sex. The aging process itself modifies the musculature via increased catabolic and decreased anabolic stimuli. Age-related hormonal changes, growth hormone, and insulin-like growth factor 1, and increased insulin resistance, together with changes in neural input are associated with decreased muscle mass. Moreover, in aging muscles, the development of sarcopenia is related to increased levels of the proinflammatory cytokines, i.e. tumor necrosis factor α and interleukin 6, and the development of mitochondrial dysfunction (14, 20, 21). In clinical practice, sarcopenia is diagnosed via HGS as a measure of muscular strength. According to the studies of patients on peritoneal dialysis and hemodialysis, HGS measurement is a good marker of nutritional status and a predictor of mortality (22, 23). In a study on patients with CKD that had not undergone dialysis, patients with lower HGS measurements progressed more rapidly to end-stage renal disease (ESRD) than others (24). In addition, HGS measurement was a better prognostic marker of renal failure than albumin (25).

Sarcopenia rarely develops in the early stage of CKD (3). A loss of muscular strength begins in the predialysis period, progresses along with the loss in kidney function, and increases morbidity (4). Patients who loss muscle strength have an increased risk of falling and fractures (26, 27). Additionally, the loss of muscle mass is more severe than expected in younger patients with CKD (4).

Many chronic conditions or diseases including end-stage organ failure, diabetes mellitus, cognitive impairment, and mood disorders are associated with chronic loss of muscle mass and strength. It has been proposed that an important factor associated with the development of sarcopenia is chronic inflammation. Moreover, the mechanism of sarcopenia development in CKD might be associated with the developing inflammatory process. Factors associated with kidney disease include nutritional deficiency, acidosis, vitamin D deficiency, calcium-phosphate metabolism disturbance, insulin resistance, diabetic nephropathy, and proteinuria. Chronic inflammation can cause a reduction in muscle mass, particularly in patients with ESRD (4). In the present study, there were no differences between the patients with and without sarcopenia regarding GFR, total protein, albumin, calcium, proteinuria, or the diabetes mellitus.

Muscle is a target organ for vitamin D. When vitamin D binds to the vitamin D receptor in skeletal muscle, muscular protein synthesis and calcium influx from cellular membranes increase. A low vitamin D level is associated with atrophy, particularly in type 2 muscle fibers, and sarcopenia (28). Although it has been demonstrated in some studies that vitamin D replacement improves muscular strength, decreases the incidence of falls, and prevents fractures, there is a lack of consensus in the literature regarding the association between the vitamin D level and muscle mass or strength (29, 30). In the present study, there was a significant positive correlation between the vitamin D level and HGS. Although univariate analysis showed that the vitamin D level was lower in patients with sarcopenia, this difference was not seen in multivariate analysis and only age was associated with sarcopenia.

The main limitation of this study was its cross-sectional design. Future prospective studies comparing sarcopenia status before and after renal transplantation are needed to better define the effect of transplantation on development of sarcopenia.

In conclusion, sarcopenia causes mobility disorders, falls, disability, poor quality of life, and even death. The present findings showed that CKD contributes to sarcopenia and sarcopenia may develop at an earlier age in RTR.
